# Evaluation of cardiometabolic risk markers linked to reduced left ventricular ejection fraction (LVEF) in patients with ST-elevation myocardial infarction (STEMI)

**DOI:** 10.1186/s12872-022-02660-3

**Published:** 2022-05-14

**Authors:** Marjan Mahdavi-Roshan, Zeinab Ghorbani, Mahboobeh Gholipour, Arsalan Salari, Amir Savar Rakhsh, Jalal Kheirkhah

**Affiliations:** 1 Cardiovascular Diseases Research Center, Department of Cardiology, Heshmat Hospital, School of Medicine, Guilan University of Medical Sciences, Rasht, Iran; 2 Department of Clinical Nutrition, School of Medicine, Guilan University of Medical Sciences, Rasht, Iran

**Keywords:** Hyperlipidemia, Overweight, Hyperglycemia, Echocardiography, Myocardial infection

## Abstract

**Background:**

It is well established that left ventricular systolic dysfunction (LVSD), as marked by reduced left ventricular ejection fraction (LVEF), notably worsens the prognosis of ST-elevation myocardial infarction (STEMI). However, the link between cardiometabolic risk markers and LVSD seems unclear. This study aimed to investigate the differences in variables affecting reduced LVEF in STEMI patients.

**Methods:**

In the current retrospective study, 200 consecutive STEMI patients were enrolled between April 2016 to January 2017. Analysis of serum parameters, anthropometric evaluation, and echocardiography was performed after admission. The participants were categorized according to LVEF levels as follows: group1 (normal: 50–70%, n = 35), group2 (mildly reduced: 40–49%, n = 48); group3 (moderately reduced: 30–39%, n = 94) and group4 (severely reduced: < 30%, n = 23). Between-group comparisons were made using the Kruskal–Wallis test.

**Results:**

Overall, of 200 STEMI patients with a mean age of 62 years, 27%(n = 54) were females. The median of BMI of patients in group4 (31.07 kg/m^2^) was significantly higher than group3 (26.35 kg/m^2^), group2 (25.91 kg/m^2^), and group1 (24.98 kg/m^2^; *P* value < 0.0001). Group4 patients showed significantly increased fasting blood sugar (FBS) than groups 1 (212.00, vs. 139.00 mg/dl; *P* value = 0.040). Patients in groups 1 and 2 exerted significantly elevated triglyceride levels than those in group4 (142.00, 142.50, and 95.00 mg/dl; *P* value = 0.001). WBC count, neutrophil%, and neutrophil to lymphocyte ratio among those in group1 (10,200/m^3^, 70.00%, and 2.92, respectively) were significantly lower than group4 (12,900/m^3^, 83.00%, and 5.47, respectively; *P* value < 0.05).

**Conclusion:**

These findings highlight higher BMI, FBS, and leucocyte count linked to LVSD, probably through increasing the inflammation and reducing LVEF levels. More extensive studies are needed to clarify the clinical relevance of these results.

## Background

Acute coronary syndrome (ACS), a major clinical presentation of atherosclerosis, has been established as the world's leading death cause [1, 2]. Myocardial ischemic states, including non-ST-elevation myocardial infarction (NSTEMI), ST-elevation myocardial infarction (STEMI), and unstable angina, are three types of ACS [3, 4]. The diagnosis and classification of ACS are made according to clinical manifestations, electrocardiogram (ECG) results, angiographic analysis, and myocardial necrosis serum markers [2, 4–6]. The currently available experimental and clinical evidence identified inflammation and the associated plaque rupture and plaque erosion, endothelial or microvascular dysfunction, and vasospasm predominantly in the epicardial arteries, as the main mechanisms involved in ACS pathogenesis [2, 5–8]. There are also additional factors including innate and adaptive immune system activation, coronary blood flow, and increased count of leukocytes (including neutrophils to lymphocytes counts ratio), that could affect the progression of ACS [2, 5–8]. Additionally, hypertension, hyperglycemia, hyperlipidemia, obesity, elevated pro-inflammatory cytokines, chronic kidney impairment, and other cardiometabolic risk factors seem to be linked with many of these processes, thus increasing the risk of vascular instability and atherosclerosis [2, 5, 6, 9]. It is well documented that the ventricular systolic dysfunction during ACS is an important clinical manifestation leading to worse outcomes [10–12]. On the other hand, it is worth noting that left ventricular ejection fraction (LVEF), determined by echocardiography, could be applied to clarify left ventricular systolic dysfunction (LVSD). Moreover, evaluation of LVEF at any time point during hospitalization has been proposed as an applicable predictor of survival in patients with ACS. In particular, LVEF less than 40% can be considered an indicator of impaired ventricular systolic function and a determinant of STEMI related mortality [11, 13–15]. Decreased LVEF that may occur because of contractile function disruption is also thought to be a strong determinant of clinical outcomes such as sustained arrhythmias of ventricles, mortality, and hospital re-admission with heart failure among patients with cardiovascular events including acute myocardial infarction and STEMI [10–13, 15].

It is possible that the distribution of obesity and serum parameters of cardiovascular risk may substantively affect STEMI progression, probably in association with impaired systolic function [16–20]. Therefore, to prevent the progression of STEMI and improve its prognosis, it would be beneficial to explore the determinants of reduced LVEF after an ACS. However, the existing literature lacks clarity regarding the association between LVEF as a measure of ventricular systolic function, upon-admission serum parameters, and body mass index (BMI) in STEMI. The current research, thus, was aimed to describe the frequencies and distributions of BMI classification, various cardiometabolic markers, and leukocyte count across LVEF categories and discuss their probable clinical relevance to impaired ventricular systolic function in a group of STEMI subjects through a cross-sectional study.

## Methods and materials

### Study population

In the current retrospective cross-sectional study, 200 patients diagnosed with STEMI referred to a tertiary referral heart center (Dr. Heshmat Cardiology Hospital) in Rasht, Iran, and underwent angiography during the hospitalization period between April 2016 to January 2017 were enrolled. Also, a number of samples were collected using the myocardial infarction (MI) patients’ registration system (MI registry). If the required information was complete, the sample was included in the study. Expert cardiologists diagnosed STEMI based on electrocardiography, physical examination, and representative symptoms of myocardial ischemia (e.g., palpitation, syncope, shortness of breath, or generalized weakness). Also, cardiac enzyme analysis was used to confirm the presence of myocardial injury and the initial diagnosis of STEMI. STEMI was defined based on the latest guidelines as the presence of ischemic chest pain (for at least 30 min) accompanying by ST-segment elevation in ≥ 2 contiguous leads (higher than 2 mm for chest derivations and higher than 1 mm for limb derivation), and myocardial ischemia and necrosis in a clinical setting [21]. Exclusion criteria were as follows: previous history of MI, NSTEMI, prior history of revascularization, hepatic, thyroid or adrenal dysfunction, familial hypercholesterolemia, intraventricular conduction disturbances, valvular diseases, hypertrophic, dilated or restrictive cardiomyopathies, dying before or during angiography or developing complications during angiography.

The institutional review board of the Cardiovascular Diseases Research Center, Guilan University of Medical Sciences, approved the research protocol (research number = 1745). Ethics committee approval was also obtained from Guilan university of medical sciences (ethic code = IR.GUMS.REC.1398.527). Also, written informed consent was collected from all patients prior to the study entry.

### Data collection

A twelve-lead electrocardiogram at rest was performed immediately after the patients' admission to the emergency room by a trained nurse. Experienced interventional cardiologist performed coronary angiography for patients via the femoral or radial artery approach. With the purpose of collecting data for the current research, two cardiologists blinded to the patients' information rechecked and interpreted the coronary angiographies. An acute thrombotic occlusion (total or subtotal) in any vessel was identified as the culprit coronary artery of ACS. Disagreements were resolved after re-examination and discussion between the two cardiologists or, if necessary, by a third interventional cardiologist. Major cardiovascular outcomes after coronary angioplasty, the burden of coronary artery disease and mortality was predicted by SYNTAX score II, which takes into account the location and characteristics of the lesion as well as demographic and clinical variables [22].

Patients' demographic and clinical data, including age, gender, smoking status, marital status, history of medication consumption and chronic disorders, in addition to the data on revascularization methods, and the drugs prescribed following revascularization were gathered. Also, anthropometric indices were collected from each patient when presenting with STEMI. BMI was then calculated as weight (kg) divided by the height (in the square, m^2^). Since a number of the cases were included from MI registry, the anthropometric indices required for calculating BMI were available in about 160 out of 200 patients. The categorization of patients based on BMI was performed according to the National Heart, Lung, and Blood Institute (NHLBI) criteria as follows: (1) normal weight (BMI between 18.5 and 24.99 kg/m^2^), overweight (BMI of 25–29.99 kg/m^2^), and obese patients (BMI of > 30 kg/m^2^).

### Hematological measurements

Fasting blood samples from each patient were collected during the first 24-h admission to the emergency room or coronary care unit. Laboratory assessments including lipid markers (i.e. total cholesterol (mg/dL), triglyceride (mg/dL), high-density lipoprotein cholesterol (HDL-C; mg/dL) and low-density lipoprotein cholesterol (LDL-C; mg/dL)), fasting blood sugar (FBS; mg/dL), leucocyte count (including white blood cell (WBC) (109 ⁄L), neutrophil count (%), lymphocyte count (%), hemoglobin (g/dL), and neutrophil to lymphocyte ratio (NLR)) analysis were carried out applying the standard methods. Among those with a history of diabetes (n = 62), fasting serum Hemoglobin A1C (HbA1c) levels were also evaluated. The myocardial necrosis biochemical markers (i.e., troponin, and creatine kinase (CK)-MB) were also assessed.

### Echocardiographic analyses

Left ventricular systolic function was assessed based on the fraction of the end-diastolic blood volume, which is ejected with each heart-beat determined with echocardiographic reports obtained within 72 h of hospital admission applying a standard commercial ultrasound machine. LVEF estimation was performed by a certified echocardiographer and confirmed by two cardiologists on the basis of the international Simpson method. The studied subjects were categorized into four groups based on their LVEF according to the 2014 American College of Cardiology heart failure guidelines, as follows: LVEF between 50 and 70% was considered normal that indicates the normal systolic function, LVEF 40% to 49% was considered mildly reduced, which means as mild left ventricular systolic dysfunction (LVSD), LVEF 30% to 39% was considered moderately reduced LVEF that indicates moderate LVSD, and LVEF less than 30% was considered severely reduced LVEF that shows severe LVSD [23].

### Statistical analysis

The Statistical Package for Social Science (SPSS for Windows, version 24.0, Chicago, IL, USA) was used for data analyses. All P-values were two-tailed, and a statistically significant difference was accepted at *P* < 0.05. The normal distribution of data was analyzed using the Kolmogorov–Smirnov test. Skewed data were reported as median (interquartile range, IQR), and the normal variable was expressed as mean (standard deviation, SD). Qualitative data were shown as numbers (percentages). Between-group comparisons were made using the Kruskal–Wallis test (or one-way analysis of variance (ANOVA)) and chi-square test for quantitative and qualitative variables, respectively. Accordingly, after significant results of these tests, to explore any significant differences between the groups two-by-two, the post hoc tests were performed.

## Results

### Characteristics of STEMI population according to left ventricular ejection fraction

Overall, a total of 200 patients (n = 54, 27% female) with STEMI with a mean (SD) age of 62 (12) years were included in the current research. The patients were assigned to four categories based on LVEF. About 35 (17.5%) STEMI subjects had normal systolic function (LVEF 50–70%), 48 (24.0%) had mild (mildly reduced LVEF 40–49%), 94 (47.0%) had moderate (moderately reduced LVEF 30–39%), and 23 (11.5%) patients had severe systolic dysfunction (severely reduced LVEF less 30% than). The characteristics of the studied population, according to LVEF, are presented in Table 1. The distribution of demographic, and past medical history data of the studied patients were not statistically different between LVEF categories. Also, the use of medications on admission was similar between the studied groups except for using insulin injection, which was significantly more prevalent among those with severely reduced LVEF (*P* value = 0.034). A HbA1c analysis was performed in 62 patients with a history of diabetes on admission, of whom 16 (25.8%) patients had HbA1c levels higher than 7%, which indicated that they might exhibit poor glycemic control.

**Table 1:** Baseline characteristics of ST-Elevation Myocardial Infarction Patients According to Left Ventricular Ejection Fraction (LVEF)

Variable	LVEF categories				
	Normal (n = 35)	Mildly reduced (n = 48)	Moderately reduced (n = 94)	Severely reduced (n = 23)	*P* value
Age (year)	57 (14)	61 (12)	63 (12)	62 (10)	0.062
Number (%) of men	27 (77.1%)	36 (75.0%)	68 (72.3%)	15 (65.2%)	0.770
*Past medical history*					
Coronary artery disease	10 (28.6%)	18 (37.5%)	37 (39.4%)	11 (47.8%)	0.504
Hypertension	19 (54.3%)	20 (41.7%)	48 (51.1%)	15 (65.2%)	0.298
Diabetes mellitus	8 (22.9%)	13 (27.1%)	31 (33.0%)	10 (43.5%)	0.351
Smoking	14 (40.0%)	22 (45.8%)	42 (44.7%)	6 (26.1%)	0.589
*Medication on admission*					
Statins	30 (85.7%)	39 (81.3%)	63 (67.0%)	16 (69.6%)	0.093
Aspirin	11 (31.4%)	23 (47.9%)	48 (51.1%)	14 (60.9%)	0.125
ARBs	14 (40.0%)	13 (27.1%)	34 (36.2%)	10 (43.5%)	0.484
ACE inhibitors	5 (14.3%)	4 (8.3%)	11 (11.7%)	3 (13.0%)	0.850
CCBs	5 (14.3%)	5 (10.4%)	14 (14.9%)	3 (13.0%)	0.903
Beta blockers	4 (11.4%)	3 (6.3%)	5 (5.3%)	1 (4.3%)	0.616
Thiazide diuretics	1 (2.9%)	3 (6.3%)	5 (5.3%)	1 (4.3%)	0.911
Anticoagulants	1 (2.9%)	1 (2.1%)	6 (6.4%)	2 (8.7%)	0.525
Metformin and/or Sulfonylureas	6 (17.1%)	10 (20.8%)	22 (23.4%)	4 (17.4%)	0.844
Insulin injection	2 (5.7%)	3 (6.3%)	8 (8.5%)	6 (26.1%)	0.034

*LVEF* left ventricular ejection fraction, *STEMI* ST elevation myocardial infarction, *ARBs* Angiotensin II receptor blockers, *ACE inhibitors* Angiotensin-converting enzyme inhibitors, *CCB* calcium channel blocker

Data of all variables are presented as number (%) except for age which is reported as mean (standard deviation)

### Clinical manifestations, angiographic characteristics, and revascularization

As summarized in Table 2, patients in different categories of LVEF presented similar clinical features related to STEMI. Patients with normal systolic function or mildly or moderately reduced LVEF predominantly had the mild type of mitral valve regurgitation in a higher proportion than those with severely reduced LVEF (*P* value = 0.001) (Table 2). Besides, all studied patients had a positive cTnI and an elevated CK–MB level (> 25 IU/L). Further, no significant between-group differences were noted in the time delay from symptom onset to presentation and the time between the first medical contacts to first device activation (FMC to FDA). Generally, the patients with more reduced LVEF tended to have more delays in admission to the hospital. In total, the mean (SD) of the duration of symptom onset to presentation and FMC to FDA were estimated as 265.50 (305.80) and 59.53 (31.02) minutes, respectively.

**Table 2:** Clinical manifestations, angiographic characteristics and revascularization of ST-Elevation Myocardial Infarction Patients According to Left Ventricular Ejection Fraction (LVEF)

Variable	LVEF categories				
	Normal (n = 35)	Mildly reduced (n = 48)	Moderately reduced (n = 94)	Severely reduced (n = 23)	*P* value
*Clinical manifestations of STEMI at admission*					
Chest pain	17 (48.6%)	28 (58.3%)	57 (60.6%)	15 (65.2%)	0.567
Epigastric pain	1 (2.9%)	3 (6.3%)	5 (5.3%)	1 (4.3%)	0.911
Nausea	4 (11.4%)	6 (12.5%)	17 (18.1%)	6 (26.1%)	0.405
Vomiting	1 (2.9%)	5 (10.4%)	10 (10.6%)	5 (21.7%)	0.153
Symptom onset to presentation time	120.00 (220.00)	180.00 (317.50)	180.00 (281.25)	240.00 (540.00)	0.758
FMC to FDA	60.00 (50.00)	50.00 (50.00)	50.00 (60.00)	45.00 (60.00)	0.553
*Angiographic characteristics*					
Culprit coronary artery					
LAD	17 (48.6%)	29 (60.4%)	59 (62.8%)	21 (91.3%)	0.001
LCX	14 (40.0%)	5 (10.4%)	11 (11.7%)	0 (0.0%)	
RCA	3 (8.6%)	10 (20.8%)	17 (18.1%)	1 (4.3%)	
Type of coronary disease					
No occlusion	1 (2.9%)	4 (8.3%)	7 (7.4%)	1 (4.3%)	0.181
One-vessel coronary disease	6 (17.1%)	12 (25.0%)	20 (21.3%)	1 (4.3%)	
Two-vessel coronary disease	15 (42.9%)	10 (20.8%)	24 (25.5%)	3 (13.0%)	
Three-vessel coronary disease	13 (37.1%)	22 (45.8%)	43 (45.7%)	18 (78.3%)	
SYNTAX score II					
< 22	25 (71.4%)	33 (68.8%)	64 (68.1%)	10 (43.5%)	0.111
> 22	10 (28.6%)	15 (31.3%)	30 (31.9%)	13 (56.5%)	
Mitral valve regurgitation					
Mild	30 (85.7%)	43 (89.6%)	72 (76.6%)	11 (47.8%)	0.002
Moderate	3 (8.6%)	4 (8.3%)	17 (18.1%)	5 (21.7%)	
Severe	2 (5.7%)	1 (2.1%)	5 (5.3%)	7 (30.4%)	
Revascularization					
Fibrinolytics	8 (22.9%)	16 (33.3%)	31 (33.0%)	5 (21.7%)	0.522
Primary PCI	27 (77.1%)	32 (66.7%)	63 (67.0%)	18 (78.3%)	
Medication prescribed at discharge					
Antiplatelets	35 (100.0%)	48 (100.0%)	94 (100.0%)	23 (100.0%)	–
Statins	35 (100.0%)	48 (100.0%)	94 (100.0%)	23 (100.0%)	–
PPIs	35 (100.0%)	48 (100.0%)	94 (100.0%)	23 (100.0%)	–
Beta blockers	24 (68.6%)	30 (62.5%)	52 (55.3%)	12 (52.2%)	0.468
Anticoagulants	3 (8.6%)	6 (12.5%)	24 (25.5%)	4 (17.4%)	0.088

*LVEF* left ventricular ejection fraction, *STEMI* ST elevation myocardial infarction, *FMC to FDA* first medical contact to first device activation, *LAD* left anterior descending artery, *LCX* left circumflex artery, *RCA* right coronary artery, *PCI* percutaneous coronary intervention, *PPIs* proton-pump inhibitors

Data of all variables are presented as number (%) except for symptom onset to presentation time and first medical contact to first device activation (FMC to FDA) which are reported as median (interquartile range)

Regarding the angiographic characteristics of the studied subjects, it was shown that the majority of the studied STEMI population with normal to moderate systolic dysfunction (> 68%) had a SYNTAX score II of less than 22. In comparison, about 56% of the patients with severe systolic dysfunction had a Syntax score II more than 22, though no significant between group differences were found. These findings were not unexpected given that LVEF is used to calculate this SYNTAX score II.

Further, other angiographic findings showed that about half of the all STEMI patients (n = 100, 50%) were diagnosed with the three-vessel coronary disease and the left anterior descending artery (LAD) was the culprit coronary artery in about 63% of these patients. Notably, almost all of the patients with severely reduced LVEF (or LVEF < 30%) had LAD occlusions. In addition, it was found the majority of patients with severely reduced LVEF (approximately 78%, n = 18) were more likely to have multi-vessel disease (Table 2). In all studied groups, the initial approach for coronary revascularization was performed through either fibrinolytic drugs prescription (n = 30% in total) or primary PCI (n = 70% in total) based on the attending interventional cardiologists' judgment.

### BMI analysis of patients with STEMI according to LVEF categories

**Fig. 1:**
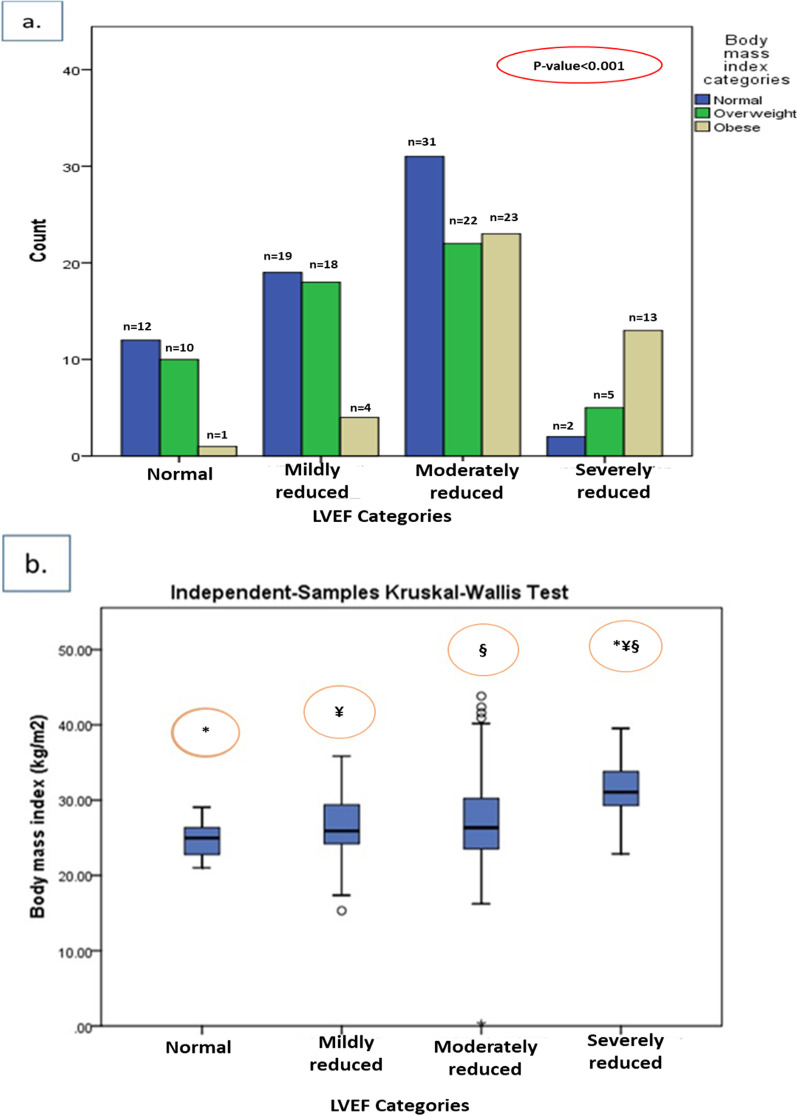
**a** Distribution of studied patients' BMI classification according to LVEF categories (Total number of included subjects = 160). **b** Comparison of median (25th and 75th percentiles) of BMI across LVEF categories (Total number of included subjects = 160). The vertical lines are the 5th and 95th percentiles of BMI. The outliers are shown by circles. BMI, body mass index; LVEF, left ventricular ejection fraction

The information on both height and weight required for BMI calculation was available in a total of 160 patients. Regarding BMI classification, the majority of patients with severely reduced LVEF (n = 13 out of 20 subjects, 65%) were more likely to be obese (BMI > 30 kg/m^2^) compared to other LVEF categories, particularly patients with normal systolic function, in which nearly half (n = 12 out of 23 subjects, 52%) of the subjects had normal BMI (*P* value < 0.001) (Fig. 1a). When analyzing BMI as a continuous variable, it was noted that BMI were significantly increasingly higher across lower LVEF categories. The median (IQR) of BMI was shown to be about 31.07 (4.84) kg/m^2^ among patients with severely reduced LVEF, which was significantly higher than that of moderately reduced (26.35 (6.75) kg/m^2^), mildly reduced (25.91 (5.31) kg/m^2^), and normal LVEF category (24.98 (3.95) kg/m^2^) (*P* value < 0.0001) (Fig. 1b).

### Serum parameters analysis of patients with STEMI according to LVEF categories

**Fig. 2:**
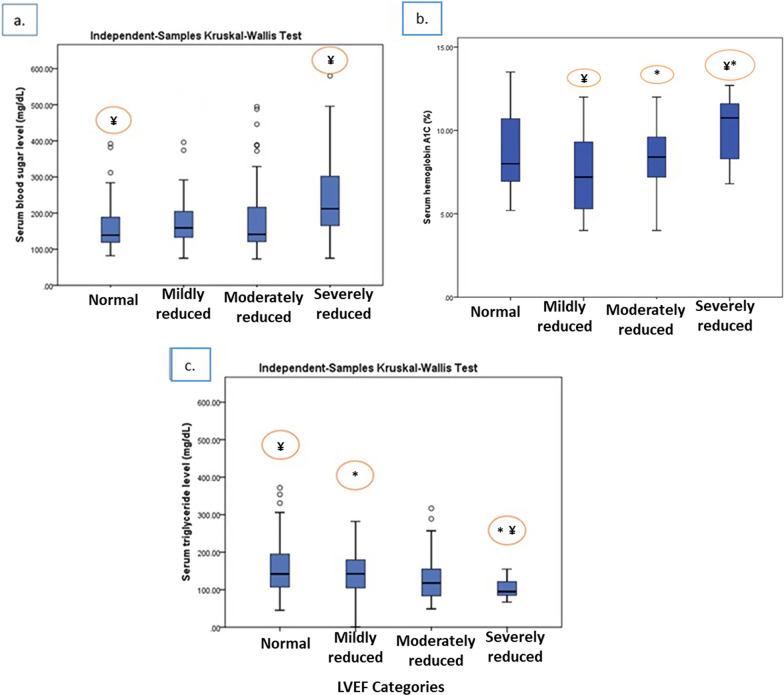
Comparison of median (25th and 75th percentiles) of blood sugar, serum Hemoglobin A1c (HbA1c, n = 62 diabetic subjects) and serum triglyceride levels across LVEF categories. The vertical lines are the 5th and 95th percentiles of serum sugar levels (**a**), serum Hemoglobin A1c (HbA1c) levels (**b**), and triglyceride levels (**c**). The outliers are shown by circles. LVEF, left ventricular ejection fraction

Kruskal–Wallis test revealed that patients across the four LVEF groups had significantly different FBS, HbA1c and triglyceride levels (Fig. 2a–c). The patients with severely reduced LVEF showed significantly elevated FBS levels (median FBS = 212.00 mg/dl) compared with the patients with normal LVEF (median FBS = 139.00 mg/dl) (*P* value = 0.040) (Table 3 and Fig. 2a). Besides, the diabetic subjects with severely reduced LVEF were also found to have significantly higher HbA1c levels (mean HbA1c = 10.22%) than the patients with mild and moderately reduced LVEF (mean HbA1c = 7.52% and 8.22%, respectively) (*P* value = 0.051) (Table 3 and Fig. 2b). Notably, except for triglyceride levels, serum lipid markers, including total cholesterol, HDL-C, and LDL-C levels, did not significantly differ across LVEF categories (Table 3 and Fig. 2c).

**Table 3:** Analysis of serum markers across Left Ventricular Ejection Fraction (LVEF) among ST-Elevation Myocardial Infarction Patients

	LVEF categories				
	Normal (n = 35)	Mildly reduced (n = 48)	Moderately reduced (n = 94)	Severely reduced (n = 23)	*P* value
Fasting blood sugar level (FBS) (mg/dL)	139.00 (70)	159.00 (73.75)	141.00 (97)	212.00 (167)	0.040
Serum Hemoglobin A1C (HbA1c) (%)^a^	8.75 (2.70)	7.52 (2.78)	8.22 (2.13)	10.22 (1.99)	0.051
High-density lipoprotein cholesterol (HDL-C; mg/dL)	38.00 (6)	38.00 (9)	38.00 (10)	37.00 (13)	0.769
Low-density lipoprotein cholesterol (LDL-C; mg/dL)	97.40 (36.80)	101.30 (51.65)	92.20 (39.40)	94.60 (49.00)	0.467
Total cholesterol (mg/dL)	172.00 (65.00)	167.00 (72.00)	156.00 (46.75)	153.00 (59.00)	0.151
Serum triglyceride level (mg/dL)	142.00 (97.00)	142.50 (78.25)	118.07 (71.75)	95.00 (41.00)	0.001
Serum WBC count/m3	10,200.00 (4500)	10,050.00 (4150)	11,500.00 (5550)	12,900.00 (4900)	0.029
Hemoglobin (g/dL)	13.42 (2.09)	12.94 (1.73)	12.74 (2.26)	12.23 (2.26)	0.189
Serum neutrophil count (%)	70.00 (18.00)	78.50 (17.00)	75.00 (16.00)	83.00 (13.00)	0.002
Serum lymphocyte count (%)	25.00 (15.00)	19.00 (16.25)	21.00 (15.00)	15.00 (13.00)	0.033
neutrophil to lymphocyte ratio (NLR)	2.92 (3.13)	4.01 (3.82)	3.57 (3.32)	5.47 (5.54)	0.021

Data of all variables are presented as median (interquartile range) except for hemoglobin and HbA1c which are reported as mean (standard deviation)

^a^HBA1c levels were only measured for those who had a history of diabetes (n = 62)

**Fig. 3:**
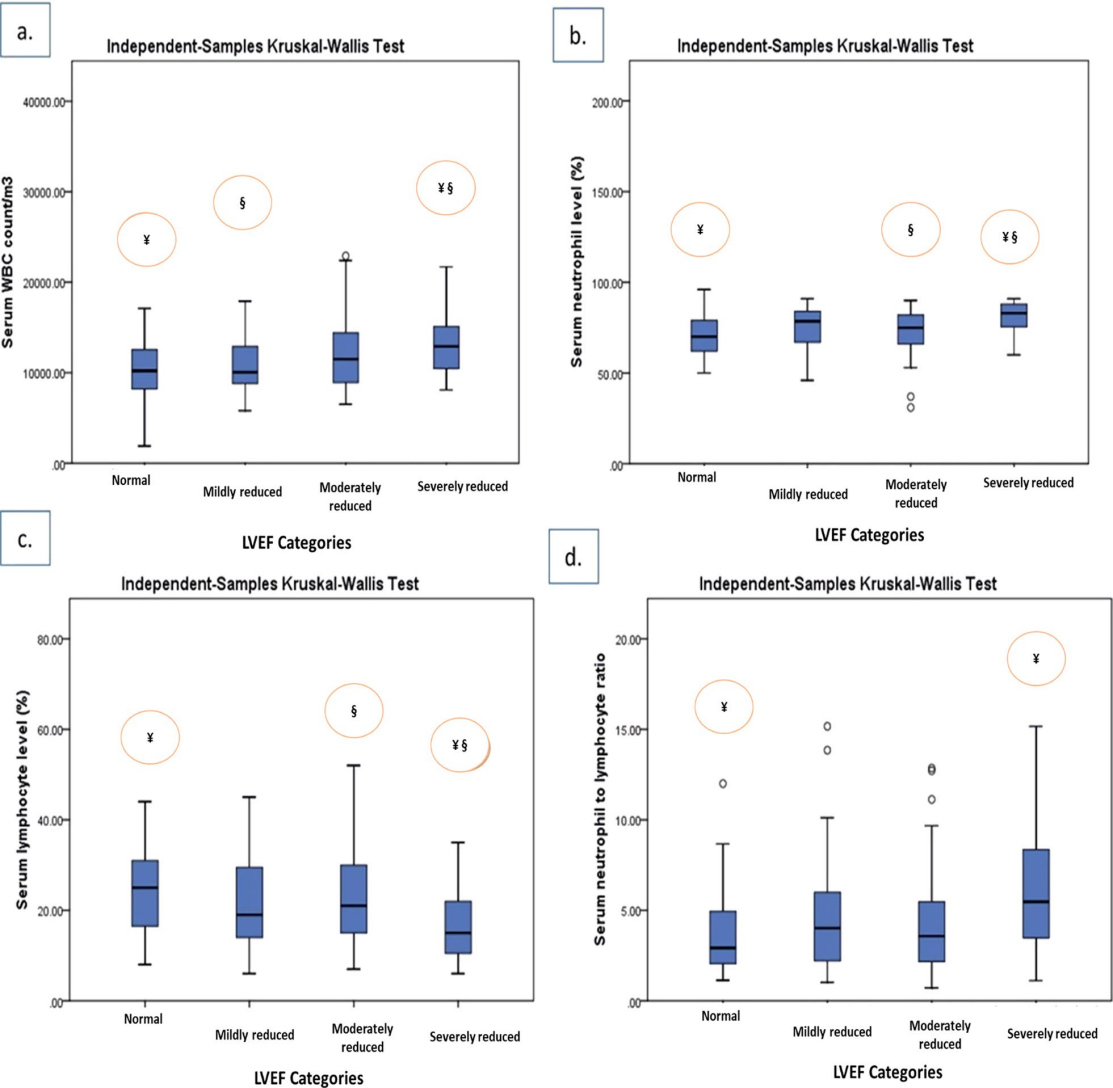
Comparison of median (25th and 75th percentiles) of serum white blood cell count and differential across LVEF categories. The vertical lines are the 5th and 95th percentiles of serum white blood counts (WBC) (**a**), neutrophil counts (**b**), lymphocyte count (**c**), and neutrophil to lymphocyte ratio (NTR) (**d**). The outliers are shown by circles. LVEF, left ventricular ejection fraction

The Kruskal–Wallis post hoc test revealed that serum WBC and neutrophil percentages as well as NLR among the STEMI patients with severely reduced LVEF levels (< 30%) (median = 12,900/m^3^, 83.00%, and 5.47, respectively) were significantly higher than those of normal LVEF group (50–70%) (median = 10,200/m^3^, 70.00%, and 2.92, respectively) (*P* value < 0.05). Also, the WBC count of the patients with severely reduced LVEF group was shown to be significantly higher than that of mildly reduced LVEF (median = 10,050/m^3^) (*P* value < 0.05). Further, the patients with severely reduced LVEF had a significantly elevated count of neutrophils than the patients in the moderately reduced LVEF category (median = 75.00%) (*P* value < 0.05). However, total lymphocyte count was indicated to be decreased among the patients in the lowest (severely reduced) LVEF category (median = 15.00%) than that of the patients in the normal and moderately reduced LVEF group (median = 25.00%, and 21.00%, respectively) (*P* value = 0.033). There are no significant differences in serum levels of hemoglobin between the patients in various LVEF groups (Table 3 and Fig. 3a–d).

## Discussion

Based on the current study findings, STEMI patients with severe LVSD as indicated by LVEF lower than 30%, were more likely to have higher BMI, showed more prevalence of overweight and obesity, and had elevated levels of FBS and leucocyte count (i.e., WBC, neutrophil percentages in addition to NLR) compa.red to those with normal systolic function as marked by LVEF of 50–70%. Of note, since almost all of the patients with severely reduced LVEF were found to have LAD occlusions, it could additionally explain the severe LVSD of this group.

The evaluation of currently identified variables could be applicable to detect high-risk STEMI patients upon admission and identify their increased need for appropriate treatments and close monitoring. However, there were paradoxical findings regarding decreased triglyceride levels in patients with severely reduced LVEF compared to those with normal LVEF.

Our results were in line with previous reports. In 2011, in a study by Jia et al. [24], 850 subjects with suspected or confirmed coronary atherosclerosis who underwent coronary angiography were included to explore LVEF level predictors. Following the present findings, they indicated that the patients in lower LVEF categories had significantly greater blood glucose and WBC count (including total leucocyte, neutrophil, and monocyte count) and lower count lymphocytes [24]. Although we failed to detect significant differences in serum HDL-C levels of the studied group, the mentioned researchers found significantly higher serum HDL-C levels among those with higher LVEF. In further analysis, they also showed significant direct associations between LVEF, HDL, and lymphocyte count [24]. Partly similar to our findings, a study by Sheeren Khaled et al. [5] on 299 ACS patients revealed that history of diabetes and kidney dysfunction was more prevalent among patients with LVEF of lower than 40% (n = 193) compared to those with LVEF of higher than 40% (n = 106). They also indicated that having a diabetes history increased the risk of LVSD by approximately 2.5 times. However, in contrast to the current results, they did not detect significant differences in BMI or serum lipid markers of the two studied groups [5]. Further, contrary to our findings, in research by Rahul Samanta et al., no differences were found in LVEF levels of normal-weight, overweight or obese patients presenting with STEMI [18].

While the effects of obesity on an accelerated burden of coronary artery disease have been well-understood [18, 25], it should be noted that the paradoxical association between BMI and survival after MI is still a matter of controversy [16–20, 25]. As it is shown in the literature, obesity per se may contribute to disturbed myocardial function. Furthermore, considering the findings in the present study, in addition to higher prevalence of obesity/overweight among the patients with severely reduced LVEF, these subjects were also reported to have a greater prevalence of coronary artery disease, hypertension, and diabetes, compared with those with normal LVEF. Thus, the association between impaired LVEF and overweight and obesity may also contribute to elevating the risk of chronic cardiometabolic dysfunction among these patients. Obesity-related cardiometabolic dysfunction could induce inflammation, oxidative stress, and myocardial injury, heighten myocardial stiffness and lower contractility that all would lead to reducing LVEF [18, 19, 25–28]. In particular, higher risk of hyperglycemia and elevated HbA1c levels in association with obesity, as it is indicated in this research, would be able to affect left ventricular mass regardless of other risk factors [18, 19, 25–29]. These findings mirror previous studies that identified hyperglycemia as a prevalent and mostly untreated comorbidity in ACS hospitalized patients, which is strongly related to poor outcomes, LVSD, renal dysfunction, and mortality in diabetic or nondiabetic subjects [2, 30–32]. Also, LVEF of diabetic subjects was shown to be lesser than those without diabetes [32]. Remarkably, elevated levels of blood sugar could augment intracellular calcium levels which in turn raise blood pressure and left ventricular mass [18, 19, 25–28]. These effects may also partly be explained by hyperglycemia-induced inflammation, hypercoagulation, platelet aggregation, cell injury, apoptosis, disturbed metabolism of ischemic myocardia, and dysfunction of endothelium in the ischemic region [2, 25, 30, 31].

The current study found no significant differences in serum lipid markers (i.e., HDL-C and LDL-C levels) across LVEF categories, except for serum triglyceride levels. Surprisingly, we demonstrated that the patients with normal and mild impaired systolic function were revealed to have significantly higher triglyceride levels than those who had a severely impaired systolic function. As mentioned, having a history of dyslipidemia is a well-established risk factor of CVDs, particularly ACS; however, aphasic fluctuations in serum lipid markers have been previously noted upon a serious cardiovascular event such as acute MI and must be considered in all patients. This phasic fluctuation refers to diminished total cholesterol, LDL-C, HDL-C, and elevated triglyceride levels [33]. In another research that enrolled STEMI (n = 212), the authors reported that there might be slight variations in mean serum levels of lipid markers during four days following ACS, which can be considered in treatment procedures, particularly when selecting the lipid-lowering drugs [34]. The current evidence could indicate that dyslipidemia's prognostic value, especially hypertriglyceridemia or hypercholesterolemia in ACS, might be complicated due to the paradoxical relationship observed between triglyceride, total cholesterol, LVEF levels, and ACS in the current and previous studies.

Moreover, our obtained results regarding increased WBC, neutrophil percentages, and NLR among those with probably severe impaired systolic function in the current study were broadly in line with previous reports that demonstrated the association between the raised levels of WBC count, particularly neutrophil counts and NLR and elevated risk of worse clinical outcomes including cardiac events and mortality in ACS subjects, especially those with STEMI [24, 35–44]. Based on research on 363 ACS patients, total leukocyte and neutrophil counts assessed one day following PCI were positively correlated with infarct size and negatively correlated with LVEF level [44]. On the other hand, regarding lymphocytes, it was reported that a higher quartile is related to a reduced mortality rate [39]. Interestingly, a systematic review of 21 studies encouraged the assessment of neutrophils (absolute or relative count, NLR) as an economical and easily acquired inflammatory indicator for stratification of risk in those who suffer from ACS and/or cardiac revascularization [45]. It has also been highlighted that increased NLR and higher leukocyte counts are independently associated with reduced EF, hospital adverse outcomes, and higher mortality rates even after 5 years of follow-up in STEMI patients [36, 41, 46–49]. Higher leukocyte counts have additionally been linked to worsened epicardial and myocardial perfusion and poor prognosis in an even more prominent manner than atherogenic lipid factors [41, 47–52]. There could be several explanations for these findings. Curiously, it has been well recognized that there is a direct relationship between leukocytes and systemic inflammation mediators such as C-reactive protein and interleukin 6 [50–52]. The observation of the raised WBC, neutrophil count, and NLR across lower LVEF categories has also been linked to intensified inflammation. Thus, the increments in leucocytes that might reflect augmented subclinical inflammatory responses, could potentially contribute to the progression and destabilization of atherosclerosis, thrombotic events as well as ischemia and disturbed coronary perfusion and reperfusion [24, 37–43, 47, 53].

### Limitation

We acknowledge that study had a number of limitations. One of the major limitations of this study was the lack of information on the success of revascularization including restoration of LVEF. Further, the retrospective, cross-sectional design might impose some limitations on results interpretation and generalizability. It also does not provide the cause-and-effect evidence between the LVEF level and the investigated variables. Moreover, although the current research was performed as a single-center study, we tried our best to ensure that all consecutive STEMI patients referred to our tertiary heart center with available data were enrolled within the determined time point. In addition, the quantitative analysis of cardiac enzymes including troponin in relation to LVEF and LVSD was lacked in this research. Lastly, the role of LAD occlusion and its correlation with LVEF, infarct size and other findings related to STEMI (like STEMI risk scores such as the Zwolle risk score (ZRS) [54]) could be an interesting topic of research for future studies in this field.

## Conclusion

The current findings highlight that patients with severe or moderate LVSD were more likely to be overweight and obese and had elevated levels of FBS and leucocyte count. These cardiometabolic risk markers might be linked to LVSD probably through augmenting the inflammatory state in association with reduced LVEF levels. Thus, evaluation of the identified variables could be applicable to detect high-risk STEMI patients upon admission. However, more extensive studies are needed to clarify the clinical relevance of the link between metabolic and anthropometric markers, and impaired left ventricular systolic function.

## Data Availability

The datasets of the current study are available from the corresponding author on reasonable request.

## Abbreviations

ACS: Acute coronary syndrome
NSTEMI: Non-ST-elevation myocardial infarction
STEMI: ST-elevation myocardial infarction
ECG: Electrocardiogram
NLR: Neutrophils to lymphocytes counts ratio
FPG: Fasting plasma glucose
LVEF: Left ventricular ejection fraction
BMI: Body mass index
PCI: Percutaneous coronary intervention
MI: Myocardial infarction
NHLBI: National Heart, Lung, and Blood Institute
HDL-C: High-density lipoprotein cholesterol
LDL-C: Low-density lipoprotein cholesterol
NLR: Neutrophil to lymphocyte ratio
IQR: Interquartile range
SD: Standard deviation
ANOVA: One-way analysis of variance
CVDs: Cardiovascular disorders
CRP: C-reactive protein
